# Soluble CD40 Ligand in Aspirin-Treated Patients Undergoing Cardiac Catheterization

**DOI:** 10.1371/journal.pone.0134599

**Published:** 2015-08-03

**Authors:** Thomas Gremmel, Andrew L. Frelinger, Alan D. Michelson

**Affiliations:** 1 Center for Platelet Research Studies, Division of Hematology/Oncology, Boston Children´s Hospital, Dana-Farber Cancer Institute, Harvard Medical School, Boston, Massachusetts, United States of America; 2 Department of Internal Medicine II, Medical University of Vienna, Vienna, Austria; University Hospital Medical Centre, GERMANY

## Abstract

Plasma soluble CD40 ligand (sCD40L) is mainly generated by cleavage of CD40L from the surface of activated platelets, and therefore considered a platelet activation marker. Although the predictive value of sCD40L for ischemic events has been demonstrated in patients with acute coronary syndromes (ACS), studies on the association of sCD40L with cardiovascular outcomes in lower risk populations yielded heterogeneous results. We therefore sought to investigate factors influencing sCD40L levels, and the predictive value of sCD40L for long-term ischemic events in unselected, aspirin-treated patients undergoing cardiac catheterization. sCD40L was determined by a commercially available enzyme-linked immunosorbent assay in 682 consecutive patients undergoing cardiac catheterization. Two-year follow-up data were obtained from 562 patients. Dual antiplatelet therapy with aspirin and clopidogrel was associated with significantly lower levels of sCD40L and lower platelet surface expressions of P-selectin and activated GPIIb/IIIa compared to aspirin monotherapy (all p≤0.01). Hypertension was linked to lower plasma concentrations of sCD40L, whereas female sex, increasing high-sensitivity C-reactive protein, and hematocrit were associated with higher sCD40L concentrations (all p<0.05). sCD40L levels were similar in patients without and with the primary endpoint in the overall study population (p = 0.4). Likewise, sCD40L levels did not differ significantly between patients without and with the secondary endpoints (both p≥0.4). Similar results were obtained when only patients with angiographically-proven coronary artery disease (n = 459), stent implantation (n = 205) or ACS (n = 125) were analyzed. The adjustment for differences in patient characteristics by multivariate regression analyses did not change the results. ROC curve analyses did not reveal cut-off values for sCD40L for the prediction of the primary or secondary endpoints. In conclusion, plasma sCD40L levels are reduced by antiplatelet therapy with clopidogrel, but not associated with long-term ischemic outcomes in unselected consecutive aspirin-treated patients undergoing cardiac catheterization.

## Introduction

Platelets are key players in atherosclerotic cardiovascular disease [1]. Following plaque rupture, activated platelets rapidly adhere to subendothelial structures of the injured vessel wall leading to intravascular thombus formation with subsequent vessel occlusion and potentially life-threatening consequences like myocardial infarction (MI). Moreover, platelets release a myriad of substances from their granules which further enhance platelet activation and aggregation as well as the interaction of platelets with leukocytes, and exert proinflammatory stimuli.

CD40 ligand (CD40L) is a trimeric, transmembrane protein and member of the tumor necrosis factor family. Following platelet activation, CD40L is rapidly expressed on the platelet surface. It then binds to CD40 on leukocytes and endothelial cells thereby inducing inflammatory responses and leukocyte-platelet aggregate formation. Soluble CD40L (sCD40L) is mainly generated by cleavage of CD40L from the surface of activated platelets [2, 3], and is therefore considered a platelet activation marker [4]. Indeed, increased levels of circulating sCD40L have been reported in many pathophysiological circumstances that are associated with platelet activation, including diabetes [5], peripheral artery disease [6], recent MI [7] and acute cerebral ischemia [8]. Several studies have investigated the association of sCD40L with ischemic outcomes in different patient populations. While high sCD40L levels were linked to increased rates of major adverse cardiovascular events (MACE) in patients with ACS [9–12], the investigation of sCD40L levels in lower risk populations has yielded heterogeneous results. Schönbeck et al. reported high plasma concentrations of sCD40L to be associated with increased vascular risk in apparently healthy women [13]. In contrast, sCD40L did not predict ischemic events in a large study of primarily healthy older men and women [14], and was not associated with subclinical atherosclerosis in a large multiethnic population from the Dallas Heart Study [15].

Data on the predictive value of sCD40L for long-term cardiovascular outcomes in unselected consecutive patients undergoing coronary angiography are missing, so far. In the current study, we therefore sought to investigate factors influencing sCD40L, and the associations of sCD40L levels with MACE in a large cohort of unselected consecutive aspirin-treated patients undergoing cardiac catheterization.

## Materials and Methods

### Study population

The study was approved by the Committee for the Protection of Human Subjects at the University of Massachusetts Medical School and has been described previously [16]. All patients presenting to the University of Massachusetts Memorial Medical Center for diagnostic cardiac catheterization for the evaluation of coronary artery disease (CAD) between the hours of 7 AM and 3 PM on weekdays from July 2002 through July 2004 were evaluated, and those who met the enrollment criteria of self-reported intake of either 81 or 325 mg of aspirin per day for ≥3 days were invited to participate. Patients receiving GPIIb/IIIa antagonists were excluded. A total of 700 consecutive patients were enrolled. Less than 3% of eligible patients declined participation. After patients provided written informed consent, blood was drawn before angiography from the femoral artery or vein after sheath insertion. Of note, pressure cuffs were not used within 30 minutes of blood sampling. The blood was immediately placed in evacuated tubes containing 3.2% sodium citrate (BD Biosciences, San Jose, California). As described previously [16], 17 patients were excluded because of blood collection issues, and 1 patient withdrew after consenting. Evaluable results were therefore obtained from 682 subjects.

### Determination of sCD40L

Citrated plasma was prepared by centrifugation and aliquots frozen at -80°C for batched analysis. Plasma sCD40L was measured using enzyme-linked immunosorbent assay kits according to the manufacturer’s instructions (R&D Systems, Minneapolis, USA). The minimum detectable dose (MDD) of human sCD40L with this assay ranges from 2.1–10.1pg/mL with a mean MDD of 4.2pg/mL. The assay is highly specific for human sCD40L, and the manufacturer reports <0.5% cross-reactivity with available related molecules.

### Platelet surface activated glycoprotein (GP) IIb/IIIa and P-selectin

Platelet surface fibrinogen receptor (GPIIb/IIIa, integrin αIIbβ3, CD41/CD61) activation and platelet surface P-selectin were measured by flow cytometry as previously described [16]. Fluorescein isothiocyanate–conjugated PAC-1 (a monoclonal antibody specific for the activated conformation of GPIIb/IIIa), phycoerythrin-conjugated CD62P (a P-selectin–specific monoclonal antibody), peridinin chlorophyll protein–conjugated CD61 (a GPIIIa-specific monoclonal antibody), and control IgG-phycoerythrin were from Becton Dickinson. Aliquots of 3.2% citrate anticoagulated blood were incubated for 5 minutes at 37°C, and then incubated with buffer for 5 minutes in the presence of PAC1-fluorescein isothiocyanate. Samples were then fixed by the addition of 1% formaldehyde in 10 mmol/L HEPES, 0.15 mol/L sodium chloride, pH 7.4. After 30 minutes’ fixation, the samples were diluted with 0.5% bovine serum albumin in 10 mmol/L HEPES, 0.15 mol/L sodium chloride (pH 7.4) and stained with CD61-peridinin chlorophyll protein (as a platelet identifier) and CD62P-phycoerythrin. Sample analysis was performed in the FACSCalibur flow cytometer with CellQuest software (Becton Dickinson), as previously described [16]. Positive analysis regions for activated GPIIb/IIIa and P-selectin, respectively, were set with appropriate nonspecific controls. Platelet surface activated GPIIb/IIIa and P-selectin were analyzed by mean fluorescence intensity (MFI).

### Clinical follow-up

Adverse clinical outcomes of all-cause death and MACE (cardiovascular death, acute coronary syndrome [ACS], hospitalization for revascularization [coronary artery bypass grafting (CABG) or percutaneous coronary intervention (PCI)], ischemic stroke or transient ischemic attack [TIA]) were assessed by telephone interview and/or medical record review. Revascularization during the index procedure at enrollment was not counted as a MACE. Follow-up contact was initiated at 18 to 24 months after enrollment; no study-related patient contact occurred between enrollment and follow-up. Patients were identified as lost to follow-up if contact was not made within 5 telephone attempts and medical records were unavailable. All-cause death was assessed for all subjects by review of medical records and the Social Security Death Index (online database) (Provo, Utah: The Generations Network, Inc, 2007. Original data: Social Security Administration, SSDI, Master File; Social Security Administration accessed via www.ancestry.com). For some individuals, cause of death was obtained from Massachusetts Registry of Vital Records and Statistics. Cause of death could not be identified for 2 patients identified as deceased through the Social Security Death Index but for whom medical records and death certificate were not available. Patients not listed as expired were presumed to be living. All clinical outcomes data were obtained by research personnel who were blinded to the results of the laboratory tests.

### Clinical endpoints

The primary endpoint was defined as the composite of the first occurrence of any of the following events within 2 years after cardiac catheterization: ACS (myocardial infarction or unstable angina), nonfatal ischemic stroke or TIA, cardiovascular death, and hospitalization for revascularization (CABG or PCI). Hospitalization for revascularization (revascularization endpoint) and the composite of the first occurrence of ACS, nonfatal ischemic stroke or TIA, and cardiovascular death (atherothrombotic endpoint) within the 2-year follow-up were defined as secondary endpoints.

### Statistical analysis

A power calculation was based on the observed mean ± SD (3.24 ± 3 ng/mL) of sCD40L in the 562 patients with completed follow-up after cardiac catheterization. We calculated that with an adverse event rate of 20% in 2 years, we were able to detect a 30% relative difference of sCD40L between patients without and with the primary endpoint with a power of 88% (using a two-sided alpha level of 0.05).

Statistical analysis was performed using the Statistical Package for Social Sciences (IBM SPSS version 22, Armonk, New York, USA). Mean ± SEM of continuous variables are shown. Categorical variables are given as numbers (%). Histograms and the Kolmogorov Smirnov test were used to test for normal distribution. Variables with skewed distribution were log-transformed for regression analyses. We performed Mann Whitney U tests to detect differences in continuous variables. The chi-square test and the Fisher’s exact test were used to detect differences in categorical variables, respectively. A multivariate linear regression model was used to assess influencing factors for sCD40L levels. Covariates for adjustment were selected on the basis of univariate analyses (p-value≤0.1), including age, sex, body mass index, prior MI, ACS, CAD, stent implantation, hypertension, hypercholesterolemia, diabetes, active smoking, hematocrit, white blood cell count (WBC), platelet count, high-sensitivity C-reactive protein (hsCRP), aspirin dose, use of clopidogrel, statins, angiotensin converting enzyme (ACE) inhibitors, beta-blockers, proton pump inhibitors and calcium channel blockers (S1–S22 Figs). Hypertension was defined as resting blood pressure ≥140/90 mmHg or lower values due to the intake of antihypertensive medication. Hypercholesterolemia was defined as a total cholesterol level ≥200 mg/dl or lower values due to the intake of lipid-lowering agents. Multivariate logistic regression analyses were used to adjust for patient characteristics that were different between patients without and with the primary and secondary endpoints. Receiver-operating characteristic (ROC) curve analyses were used to determine the ability of sCD40L levels to distinguish between patients without and with the primary and secondary endpoints, respectively. Two-sided P-values <0.05 were considered statistically significant.

## Results

One hundred and twenty patients (17.6%) were lost to follow-up. The remaining 562 patients (82.4%) entered statistical analysis. Clinical characteristics of these patients at the time of study entry are shown in Table 1. The average time of follow-up was 24.8 ± 0.3 months.

**Table 1:** Patient characteristics of the overall study population, and of patients with aspirin monotherapy (ASA) vs. dual antiplatelet therapy (ASA+Clo).

Characteristics	Overall (n = 562)	ASA (n = 371)	ASA+Clo (n = 191)	p
Age, years	61.6 ± 0.5	62 ± 1	61 ± 1	0.2
Sex, % male	65.3	63.9	68.1	0.3
BMI, kg/m^2^	30 ± 0.3	30.2 ± 0.3	29.7 ± 0.4	0.5
**Medical history, %**				
Prior MI	23.7	20.3	30.4	0.008
Prior stroke	3.9	3.2	5.2	0.3
Hypertension	72.2	72.8	71.2	0.7
Hyperlipidemia	83.5	82.2	85.9	0.3
Diabetes mellitus	28.5	30.5	24.6	0.1
Active smoking	19.4	19.9	18.3	0.7
**Laboratory data**				
Platelet count, x 10^9^/L	229 ± 3	227 ± 3	232 ± 6	0.8
White blood cells, x 10^9^/L	7.1 ± 0.1	7.1 ± 0.1	7 ± 0.2	0.2
Hematocrit, %	39.8 ± 0.2	40 ± 0.2	39.4 ± 0.3	0.1
High-sensitivity CRP, mg/L	8 ± 0.8	7.8 ± 0.9	8.4 ± 1.6	0.4
**Reason for catheterization, %**				
Positive exercise tolerance test	37.4	41.9	28.6	0.002
Chest pain	17.2	17.6	16.4	0.7
Stable angina	17.7	17.6	18	0.9
Unstable angina	15.2	10.5	24.3	<0.001
Non-ST-segment elevation MI	6.4	4.9	9.5	0.03
ST-segment elevation MI	0.7	0.5	1.1	0.6
Other	5.4	7	2.1	0.02
**Angiography result, %**				
Coronary artery disease	81.7	80.9	83.2	0.5
Stent implantation	36.5	34.8	39.8	0.2
**Medication pre-intervention, %**				
Aspirin	100	100	100	1
Statin	68.5	63.9	77.5	0.001
ACE inhibitor	42.3	41.5	44	0.6
Beta blocker	73.8	76.5	68.6	0.04
Calcium channel blocker	23.7	21.6	27.7	0.1
Proton pump inhibitor	31	28.3	36.1	0.06

Continuous data are shown as mean ± SEM. Dichotomous data are shown as %.

Abbreviations: ACE, angiotensin converting enzyme; BMI, body mass index; CRP, C-reactive protein; MI, myocardial infarction.

**Fig 1:**
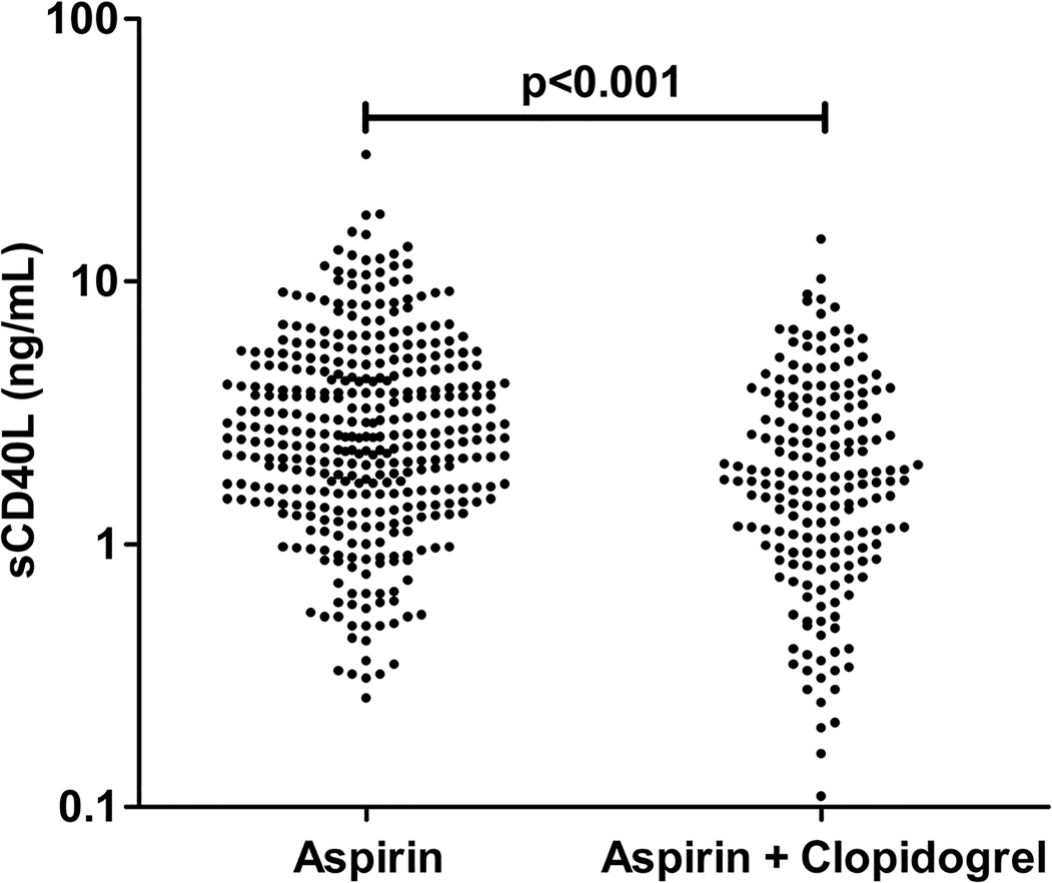
Soluble CD40 ligand (sCD40L) levels and antiplatelet therapy. Individual sCD40L levels in patients with dual antiplatelet therapy with aspirin and clopidogrel compared to patients with aspirin monotherapy.

One-hundred and seventy-six (31.3%) and 386 patients (68.7%) received low- (81mg/day) and high-dose (325mg/day) aspirin therapy, respectively. In all patients on dual antiplatelet therapy (n = 191), clopidogrel was started before angiography. Patients who had already received clopidogrel ≥3 days before angiography (n = 127; 66.5%) received 75mg clopidogrel/day, and those who had not received clopidogrel ≥3 days before angiography (n = 64; 33.5%) received a loading dose of 300mg clopidogrel followed by 75mg clopidogrel/day. The mean time interval from the last ingestion of clopidogrel until cardiac catheterization was 4.2 ± 0.3 hours. All remaining medications had been started ≥3 days before angiography. Patients with dual antiplatelet therapy had unstable angina, non ST-segment MI, a history of MI and concomitant statin therapy more often than patients with aspirin monotherapy. On the other hand, patients with aspirin monotherapy had a positive exercise tolerance test and concomitant beta blocker therapy more often than patients with dual antiplatelet therapy (all p<0.05; Table 1). sCD40L levels did not differ significantly between patients with low- and high-dose aspirin therapy (3.69 ± 0.29 ng/mL *vs*. 3.04 ± 0.13 ng/mL; p = 0.2), but were significantly lower in patients concomitantly taking clopidogrel (n = 191, 34%) compared to patients with aspirin monotherapy (n = 371, 66%; Fig 1; 2.43 ± 0.15 ng/mL *vs*. 3.66 ± 0.17 ng/mL, p<0.001). Moreover, patients on dual antiplatelet therapy had significantly lower platelet surface expressions of P-selectin and activated glycoprotein IIb/IIIa than patients on aspirin monotherapy (P-selectin: 3.04 ± 0.04 MFI *vs*. 3.19 ± 0.03 MFI, p = 0.003; activated GPIIb/IIIa: 5.63 ± 0.14 MFI *vs*. 6.17 ± 0.12 MFI, p = 0.01). Other than the use of clopidogrel, only the use of statins was linked to lower sCD40L levels in univariate analyses (S8 Fig), whereas female sex, increasing WBC count, platelet count, and hsCRP were significantly associated with higher sCD40L levels (S4–S6 Figs, S21 Fig). The use of clopidogrel, female sex, hypertension, hematocrit and hsCRP were independently associated with sCD40L levels in the multivariate linear regression model (Table 2). Thereby, only the use of clopidogrel and hypertension were linked to lower concentrations of sCD40L, whereas female sex, increasing hsCRP and hematocrit were associated with higher sCD40L levels.

**Table 2:** Regression coefficients (B), 95% confidence intervals (CI), and p-values (p) of multivariate linear regression analysis of female sex, prior myocardial infarction (MI), active smoking, hypertension, hyperlipidemia, hematocrit, log transformed white blood cell count (log WBC), platelet count, log transformed high-sensitivity C-reactive protein (log hsCRP), and the use of clopidogrel, statins and beta blockers for log transformed soluble CD40 ligand levels (log sCD40L).

	Log sCD40L		
	B	CI	P
**Female Sex**	0.08	0.001–0.15	0.047
**Prior MI**	-0.03	-0.1–0.05	0.5
**Active smoking**	0.04	-0.04–0.13	0.3
**Hypertension**	-0.07	-0.15 –-0.003	0.04
**Hyperlipidemia**	-0.003	-0.1–0.1	0.9
**Hematocrit**	0.009	0.001–0.02	0.03
**Log WBC**	0.06	-0.22–0.33	0.7
**Platelet count**	0	-0.001–0.001	0.2
**Log hsCRP**	0.1	0.05–0.16	<0.001
**Clopidogrel**	-0.17	-0.24 –-0.11	<0.001
**Statin**	0.004	-0.08–0.09	0.9
**Beta Blocker**	0.06	-0.01–0.13	0.1

In a subanalysis of female patients, the use of clopidogrel and hypertension were independently associated with lower plasma concentrations of sCD40L, whereas hematocrit, the use of beta-blockers and increasing age were associated with higher sCD40L levels (all p<0.05).

Troponin T and creatine kinase levels were available in 123 and 238 patients, respectively, and did not correlate significantly with plasma concentrations of sCD40L (both p = 0.8).

The primary endpoint occurred in 117 patients (20.8%) and was mainly driven by revascularisation (n = 57; 10.1%) and ACS (n = 46; 8.2%). Ischemic stroke/TIA occurred in 7 patients (1.25%), and cardiovascular death occurred in 7 patients (1.25%). One MACE occurred on day 2 after enrollment and catheterization, whereas all other MACE occurred at >7 days after enrollment and catheterization. Therefore, no censoring of early MACE events was required.

Plasma sCD40L levels were similar in patients without and with the primary endpoint in the overall study population (n = 562; Table 3). Likewise, sCD40L levels did not differ significantly between patients without and with the secondary endpoints (Table 3).

**Table 3:** Soluble CD40 ligand levels (sCD40L) in patients without and with the primary and secondary endpoints in the overall study population (n = 562).

	**No primary endpoint (n = 445)**	**Primary endpoint (n = 117)**	**p**
sCD40L (ng/mL)	3.22 ± 0.13	3.32 ± 0.36	0.4
	**No revascularization (n = 505)**	**Revascularization (n = 57)**	**p**
sCD40L (ng/mL)	3.25 ± 0.13	3.14 ± 0.42	0.4
	**No atherothrombotic event (n = 502)**	**Atherothrombotic event (n = 60)**	**p**
sCD40L (ng/mL)	3.21 ± 0.13	3.49 ± 0.57	0.7

Continuous data are given as mean ± SEM.

Similar results were obtained when only patients with angiographically-proven CAD (n = 459; Table 4), stent implantation (n = 205; Table 5) or ACS (n = 125; Table 6) were analyzed.

**Table 4:** Soluble CD40 ligand levels (sCD40L) in patients without and with the primary and secondary endpoints in the subgroup of patients with angiographically-proven coronary artery disease (n = 459).

	**No primary endpoint (n = 348)**	**Primary endpoint (n = 111)**	**p**
sCD40L (ng/mL)	3.26 ± 0.16	3.38 ± 0.37	0.5
	**No revascularization (n = 406)**	**Revascularization (n = 53)**	**p**
sCD40L (ng/mL)	3.31 ± 0.16	3.14 ± 0.45	0.3
	**No atherothrombotic event (n = 401)**	**Atherothrombotic event (n = 58)**	**p**
sCD40L (ng/mL)	3.24 ± 0.15	3.59 ± 0.58	0.9

Continuous data are given as mean ± SEM.

**Table 5:** Soluble CD40 ligand levels (sCD40L) in patients without and with the primary and secondary endpoints in the subgroup of patients with stent implantation (n = 205).

	**No primary endpoint (n = 141)**	**Primary endpoint (n = 64)**	**p**
sCD40L (ng/mL)	3.08 ± 0.24	2.99 ± 0.34	0.9
	**No revascularization (n = 171)**	**Revascularization (n = 34)**	**p**
sCD40L (ng/mL)	3.02 ± 0.21	3.21 ± 0.54	0.9
	**No atherothrombotic event (n = 175)**	**Atherothrombotic event (n = 30)**	**p**
sCD40L (ng/mL)	3.11 ± 0.22	2.75 ± 0.4	0.8

Continuous data are given as mean ± SEM.

**Table 6:** Soluble CD40 ligand levels (sCD40L) in patients without and with the primary and secondary endpoints in the subgroup of patients with acute coronary syndrome (n = 125).

	**No primary endpoint (n = 96)**	**Primary endpoint (n = 29)**	**p**
sCD40L (ng/mL)	3.22 ± 0.3	2.36 ± 0.37	0.3
	**No revascularization (n = 117)**	**Revascularization (n = 8)**	**p**
sCD40L (ng/mL)	3.03 ± 0.26	2.86 ± 0.61	0.6
	**No atherothrombotic event (n = 104)**	**Atherothrombotic event (n = 21)**	**p**
sCD40L (ng/mL)	3.19 ± 0.28	2.17 ± 0.46	0.1

Continuous data are given as mean ± SEM.

The adjustment for differences in patient characteristics by multivariate regression analyses did not change the results. ROC curve analyses did not reveal cut-off values for sCD40L levels for the prediction of the primary or secondary endpoints in the overall study population, in patients with angiographically-proven CAD, in patients undergoing stent implantation, or in ACS patients.

## Discussion

Our study is the first to investigate factors influencing sCD40L levels and the predictive value of sCD40L for 2-year MACE in a large cohort of unselected consecutive aspirin-treated patients undergoing cardiac catheterization. Dual antiplatelet therapy with aspirin and clopidogrel was independently associated with lower levels of sCD40L and lower platelet surface expressions of P-selectin and activated GPIIb/IIIa compared to aspirin monotherapy. Moreover, hypertension was linked to lower plasma concentrations of sCD40L, whereas female sex, increasing hsCRP and hematocrit were independently associated with higher sCD40L levels. We found no significant associations of sCD40L levels with 2-year ischemic outcomes in the overall study population. These findings were consistent in patients with angiographically-proven CAD, in patients undergoing stent implantation, and in ACS patients.

Our observation that clopidogrel reduces plasma concentrations of sCD40L is consistent with previous studies showing lower sCD40L levels in clopidogrel-treated CAD patients compared to patients receiving placebo [17, 18, 19]. Azar et al. randomized 73 aspirin-treated patients with stable CAD to 75mg clopidogrel/day *vs*. placebo for >6 months, and found that the addition of clopidogrel to aspirin significantly reduced sCD40L levels [17]. Similar observations were reported by Heitzer et al. and Undas et al., who randomized patients with CAD and long-term aspirin therapy to clopidogrel *vs*. placebo [18, 19]. Moreover, Yip et al. reported that the administration of a loading dose of 300mg clopidogrel substantially decreased plasma concentrations of sCD40L in patients with unstable angina, who underwent coronary stenting [20]. Likewise, a significant increase in sCD40L levels following the cessation of clopidogrel therapy in patients with CAD has been reported [21]. The assumption that dual antiplatelet therapy decreases platelet activation in the tested patient population is strengthened by our observation of signifcantly lower platelet surface expressions of P-selectin and activated GPIIb/IIIa in patients treated with aspirin and clopidogrel compared to patients on aspirin monotherapy. Further studies are needed to investigate if the beneficial effects of clopidogrel in patients with ACS are at least in part attributable to its impact on sCD40L levels.

Besides clopidogrel therapy, hypertension was associated with lower levels of sCD40L in our patient population. This may be attributable to a more intense cardiovascular risk factor management in patients with known arterial hypertension [22]. Indeed, treatment with statins, ACE inhibitors, beta blockers and calcium channel blockers was more frequent in patients with hypertension (all p≤0.003). However, none of these agents was associated with lower levels of sCD40L in the overall study population, and in a subanalysis including only patients with hypertension (all p>0.05).

The independent association of female sex with higher sCD40L concentrations may explain the increased formation of monocyte- and neutrophil-platelet aggregates in women compared to men with atherosclerotic cardiovascular disease [23]. Besides hematocrit, the use of beta-blockers and increasing age were linked to higher sCD40L levels in female patients in our study. It has been shown that the ovarian cycle has an impact on platelet activation [24]. However, the mean age of female patients of our study population was 63 ±1 years, and only 26 women (13.3%) were younger than 50 years. Therefore, most female patients in our study population can be considered postmenopausal. The association of increasing hsCRP levels with higher plasma concentrations of sCD40L was expected since sCD40L, like hsCRP, can be considered a marker of inflammation [25]. However, the role of sCD40L in inflammation is still under investigation. Elevated levels of sCD40L were found in various diseases associated with chronic inflammation including diabetes [5], atherosclerotic cardiovascular disease [6, 7], obesity [26] and inflammatory bowel disease [27]. Furthermore, the treatment of wild type CD40 mice with sCD40L induced a pulmonary inflammatory response that was not seen in identically treated CD40 knockout mice [28]. Hristov et al. reported that sCD40L impairs the function of peripheral blood angiogenic outgrowth cells and increases neointimal formation in a murine model of arterial injury [29]. On the other hand, Henn et al. showed that, in contrast to platelet-bound CD40L [25, 30], sCD40L cannot induce an inflammatory reaction of endothelial cells [2]. Consequently, it still remains to be established whether sCD40L is itself pro-inflammatory or is just a surrogate marker of ongoing inflammatory processes.

Various studies reported an association between high sCD40L concentrations and an increased risk of MACE in patients with ACS [9–12]. Pusuroglu et al. observed a significantly higher in-hospital and 1-year all-cause mortality in patients with ST-segment elevation MI (STEMI) with high sCD40L levels at hospital admission compared to STEMI patients with lower plasma concentrations of sCD40L [9]. Dominguez-Rodriguez et al. reported increased serum levels of sCD40L in STEMI patients, who subsequently developed in-hospital MACE [10]. In a nested case-control study, Varo et al. showed that elevated plasma levels of sCD40L identify patients with ACS at heightened risk of death and recurrent MI [11]. Interestingly, Morrow et al. did not report an association between high sCD40L levels and 30-day clinical outcomes in 1524 patients with ACS [31]. This discrepancy compared to the results of previous studies may be explained by the use of the glycoprotein (GP) IIb/IIIa inhibitor tirofiban in all patients of their study population. Since GPIIb/IIIa antagonists inhibit the release of CD40L from the platelet surface [32, 33], the administration of tirofiban during ACS may affect the association of sCD40L levels with short-term MACE. Indeed, Heeschen et al. showed that treatment of ACS patients with increased sCD40L levels with the GPIIb/IIIa inhibitor abciximab reduces their risk of MACE at 6 months, whereas no significant treatment effect of abciximab was found in ACS patients with low sCD40L levels [12]. It remains to be established whether GPIIb/IIIa inhibitors at the time of ACS also reduce long-term ischemic outcomes in patients with initially elevated sCD40L levels.

In contrast to the above findings in ACS patients, high serum concentrations of sCD40L were not associated with an increased risk of ischemic stroke or coronary events in a nested case-control study including 233 stable patients with CAD, who subsequently developed MACE, and 233 age- and gender-matched CAD patients without ischemic events [34]. Likewise, Rondina et al. assessed sCD40L levels in 303 patients with CAD, who developed a cardiac event within one year, in 303 patients with CAD free of MACE, and in 303 patients without CAD and free of events [35]. They also did not observe a higher risk of ischemic events in patients with elevated sCD40L. The results of the present study are consistent with these previous findings since most of our patients had stable CAD. However, our study is the first to investigate the predictive value of sCD40L for 2-year MACE in unselected consecutive aspirin-treated patients undergoing cardiac catheterization. The fact that we did not observe an association between sCD40L levels and ischemic outcomes in the subgroup of ACS patients may be attributable to the sample size. Only 125 patients (22.3%) of our study population underwent cardiac catheterization because of an ACS. Another explanation for the missing association between sCD40L levels and MACE in these patients could be the pre-treatment with aspirin for ≥3 days, since low-dose aspirin significantly reduces levels of sCD40L [36]. Of note, sCD40L levels did not differ significantly between patients with low- versus high-dose aspirin therapy in our study population. It has been shown that platelet cyclooxygenase activity, as reflected by serum thromboxane B2 levels, is uniformly suppressed by 99% of baseline by low-dose aspirin therapy [37]. Moreover, high-dose aspirin was not associated with a decreased rate of ischemic events compared to low-dose aspirin in ACS patients undergoing PCI [38]. These findings suggest that aspirin already exerts the major part of its antiplatelet effect at a low dose, which may explain why we did not observe an influence of aspirin dosage on sCD40L levels. Indeed, our study is in line with a previous report showing no effect of doubling the aspirin dosage on concentrations of sCD40L [39]. Finally, most of our patients received intense cardiovascular risk factor management, which may have affected sCD40L levels and their association with ischemic outcomes [22]. Considering the results of previous studies and our findings, sCD40L may play a role in the assessment of future cardiovascular risk in patients with MI, but seems to be of limited value in healthy individuals and in patients with stable CAD.

It has been previously shown that sCD40L levels are higher in serum than in plasma samples [40, 41]. This may affect the current data by limiting the overall amplitude of sCD40L. However, plasma sCD40L has already been associated with adverse ischemic outcomes in high risk patients [9, 11]. The lack of a predictive value of plasma sCD40L for MACE in our study is therefore likely to be mainly due to the overall lower risk profile of our patient population.

sCD40L primarily originates from platelets, but also originates from T-cells and other leukocytes [25, 42]. Although it is not possible to differentiate between platelet-derived and leukocyte-derived sCD40L, due to their number in human whole blood, platelets can be considered the major source of sCD40L. In our study, we aimed to investigate the predictive value of total sCD40L for MACE, and did not specifically address the role of platelets and leukocytes in sCD40L generation.

A limitation of our study is its observational design. Clopidogrel therapy before angiography was not randomized, but administered at the treating physician’s discretion. As expected, pretreatment with clopidogrel was used more frequently in patients with ACS (unstable angina and MI) and in those with a history of MI. We measured sCD40L levels at only one time point before cardiac catheterization. Therefore, we were not able to investigate variations of sCD40L over time and the effects of therapeutic interventions on sCD40L levels. Markers of renal function are not available for the study population. However, none of the patients had known renal failure or exhibited clinical signs of renal failure. In order to study the predictive value of sCD40L for MACE in the real-life scenario of an all-comers population, we enrolled unselected, consecutive aspirin-treated patients undergoing coronary angiography. Consequently, blood sampling was performed at various time points between 7 AM and 3 PM and not exclusively under fasting conditions. Finally, 120 patients (17.6%) were lost to follow-up, which constitutes a rather high drop out rate.

In conclusion, sCD40L levels are reduced by antiplatelet therapy with clopidogrel, but not associated with long-term ischemic outcomes in unselected aspirin-treated patients undergoing cardiac catheterization. The predictive value of sCD40L for MACE may be limited to high-risk cardiovascular populations.

## Supporting Information

S1 Dataset Data set showing soluble CD40 ligand (sCD40L) levels and patient characteristics as well as clinical endpoints of all patients with completed follow-up. (XLSX)

S1 Fig Correlation of soluble CD40 ligand (sCD40L) levels with age. Scatter plot showing sCD40L levels (x-axis) vs. age (y-axis). Circles represent individual measurements.
(PDF)

S2 Fig Correlation of soluble CD40 ligand (sCD40L) levels with body mass index (BMI). Scatter plot showing sCD40L levels (x-axis) vs. BMI (y-axis). Circles represent individual measurements.
(PDF)

S3 Fig Correlation of soluble CD40 ligand (sCD40L) levels with hematocrit. Scatter plot showing sCD40L levels (x-axis) vs. hematocrit (y-axis). Circles represent individual measurements.
(PDF)

S4 Fig Correlation of soluble CD40 ligand (sCD40L) levels with white blood cell count (WBC). Scatter plot showing sCD40L levels (x-axis) vs. WBC (y-axis). Circles represent individual measurements.
(PDF)

S5 Fig Correlation of soluble CD40 ligand (sCD40L) levels with platelet count. Scatter plot showing sCD40L levels (x-axis) vs. platelet count (y-axis). Circles represent individual measurements.
(PDF)

S6 Fig Correlation of soluble CD40 ligand (sCD40L) levels with high-sensitivity C-reactive protein (hsCRP). Scatter plot showing sCD40L levels (x-axis) vs. hsCRP (y-axis). Circles represent individual measurements.
(PDF)

S7 Fig Soluble CD40 ligand (sCD40L) levels in patients without and with clopidogrel therapy. Box plot showing sCD40L levels in patients without and with clopidogrel. The boundaries of the box show the lower and upper quartile of data, the line inside the box represents the median. Whiskers are drawn from the edge of the box to the highest and lowest values that are outside the box but within 1.5 times the box length.
(PDF)

S8 Fig Soluble CD40 ligand (sCD40L) levels in patients without and with statins. Box plot showing sCD40L levels in patients without and with statins. The boundaries of the box show the lower and upper quartile of data, the line inside the box represents the median. Whiskers are drawn from the edge of the box to the highest and lowest values that are outside the box but within 1.5 times the box length.
(PDF)

S9 Fig Soluble CD40 ligand (sCD40L) levels in patients without and with beta blockers. Box plot showing sCD40L levels in patients without and with beta blockers. The boundaries of the box show the lower and upper quartile of data, the line inside the box represents the median. Whiskers are drawn from the edge of the box to the highest and lowest values that are outside the box but within 1.5 times the box length.
(PDF)

S10 Fig Soluble CD40 ligand (sCD40L) levels in patients without and with proton pump inhibitors (PPIs). Box plot showing sCD40L levels in patients without and with PPIs. The boundaries of the box show the lower and upper quartile of data, the line inside the box represents the median. Whiskers are drawn from the edge of the box to the highest and lowest values that are outside the box but within 1.5 times the box length.
(PDF)

S11 Fig Soluble CD40 ligand (sCD40L) levels in patients without and with calcium-channel blockers (CCBs). Box plot showing sCD40L levels in patients without and with CCBs. The boundaries of the box show the lower and upper quartile of data, the line inside the box represents the median. Whiskers are drawn from the edge of the box to the highest and lowest values that are outside the box but within 1.5 times the box length.
(PDF)

S12 Fig Soluble CD40 ligand (sCD40L) levels in patients without and with angiotensin-converting enzyme inhibitors (ACEIs). Box plot showing sCD40L levels in patients without and with ACEIs. The boundaries of the box show the lower and upper quartile of data, the line inside the box represents the median. Whiskers are drawn from the edge of the box to the highest and lowest values that are outside the box but within 1.5 times the box length.
(PDF)

S13 Fig Soluble CD40 ligand (sCD40L) levels in patients without and with hypertension. Box plot showing sCD40L levels in patients without and with hypertension. The boundaries of the box show the lower and upper quartile of data, the line inside the box represents the median. Whiskers are drawn from the edge of the box to the highest and lowest values that are outside the box but within 1.5 times the box length.
(PDF)

S14 Fig Soluble CD40 ligand (sCD40L) levels in patients without and with hyperlipidemia. Box plot showing sCD40L levels in patients without and with hyperlipidemia. The boundaries of the box show the lower and upper quartile of data, the line inside the box represents the median. Whiskers are drawn from the edge of the box to the highest and lowest values that are outside the box but within 1.5 times the box length.
(PDF)

S15 Fig Soluble CD40 ligand (sCD40L) levels in patients without and with diabetes. Box plot showing sCD40L levels in patients without and with diabetes. The boundaries of the box show the lower and upper quartile of data, the line inside the box represents the median. Whiskers are drawn from the edge of the box to the highest and lowest values that are outside the box but within 1.5 times the box length.
(PDF)

S16 Fig Soluble CD40 ligand (sCD40L) levels in non-smokers and smokers. Box plot showing sCD40L levels in non-smokers and smokers. The boundaries of the box show the lower and upper quartile of data, the line inside the box represents the median. Whiskers are drawn from the edge of the box to the highest and lowest values that are outside the box but within 1.5 times the box length.
(PDF)

S17 Fig Soluble CD40 ligand (sCD40L) levels in patients without and with prior myocardial infarction (MI). Box plot showing sCD40L levels in patients without and with prior MI. The boundaries of the box show the lower and upper quartile of data, the line inside the box represents the median. Whiskers are drawn from the edge of the box to the highest and lowest values that are outside the box but within 1.5 times the box length.
(PDF)

S18 Fig Soluble CD40 ligand (sCD40L) levels in patients without and with coronary artery disease (CAD). Box plot showing sCD40L levels in patients without and with CAD. The boundaries of the box show the lower and upper quartile of data, the line inside the box represents the median. Whiskers are drawn from the edge of the box to the highest and lowest values that are outside the box but within 1.5 times the box length.
(PDF)

S19 Fig Soluble CD40 ligand (sCD40L) levels in patients without and with stent implantation. Box plot showing sCD40L levels in patients without and with stent implantation. The boundaries of the box show the lower and upper quartile of data, the line inside the box represents the median. Whiskers are drawn from the edge of the box to the highest and lowest values that are outside the box but within 1.5 times the box length.
(PDF)

S20 Fig Soluble CD40 ligand (sCD40L) levels in patients without and with acute coronary syndromes (ACS). Box plot showing sCD40L levels in patients without and with ACS. The boundaries of the box show the lower and upper quartile of data, the line inside the box represents the median. Whiskers are drawn from the edge of the box to the highest and lowest values that are outside the box but within 1.5 times the box length.
(PDF)

S21 Fig Soluble CD40 ligand (sCD40L) levels in male and female patients of the study population. Box plot showing sCD40L levels in male and female patients patients of the study population. The boundaries of the box show the lower and upper quartile of data, the line inside the box represents the median. Whiskers are drawn from the edge of the box to the highest and lowest values that are outside the box but within 1.5 times the box length.
(PDF)

S22 Fig Soluble CD40 ligand (sCD40L) levels in patients with low-dose and high-dose aspirin therapy. Box plot showing sCD40L levels in patients with low-dose and high-dose aspirin therapy. The boundaries of the box show the lower and upper quartile of data, the line inside the box represents the median. Whiskers are drawn from the edge of the box to the highest and lowest values that are outside the box but within 1.5 times the box length.
(PDF)
