# Supplementary figures and images for: Soluble CD40 Ligand in Aspirin-Treated Patients Undergoing Cardiac Catheterization
Source: PLoS One. 2015 Aug 3;10(8):e0134599. doi: 10.1371/journal.pone.0134599 (PMC4523196; doi:10.1371/journal.pone.0134599)

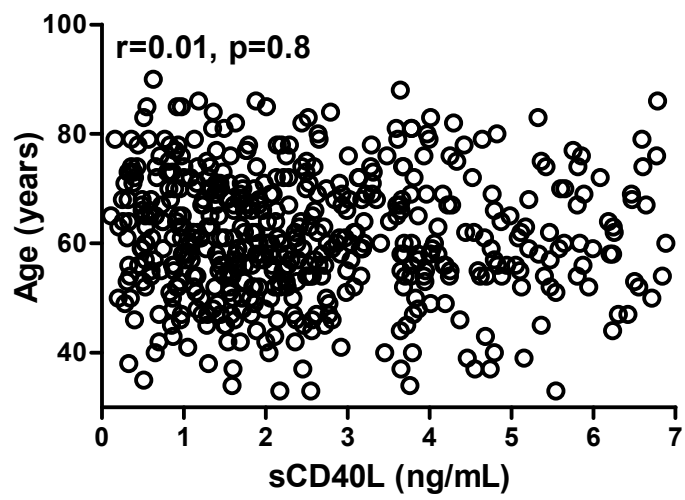

Supplement: S1 Fig — Scatter plot showing sCD40L levels (x-axis) vs. age (y-axis). Circles represent individual measurements. (PDF) [file pone.0134599.s002.pdf]

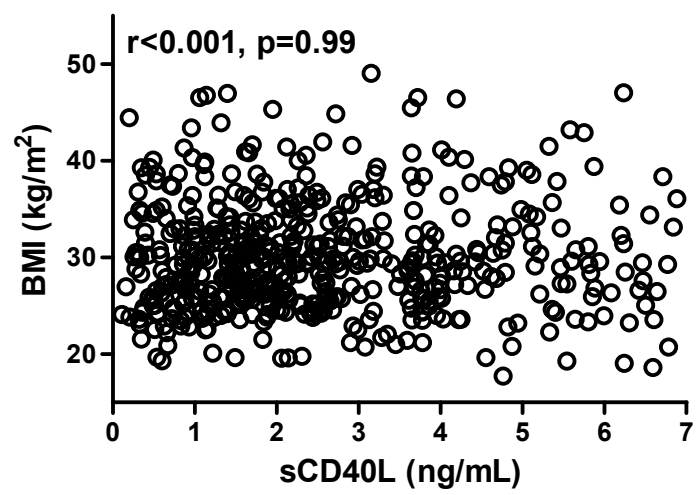

Supplement: S2 Fig — Scatter plot showing sCD40L levels (x-axis) vs. BMI (y-axis). Circles represent individual measurements. (PDF) [file pone.0134599.s003.pdf]

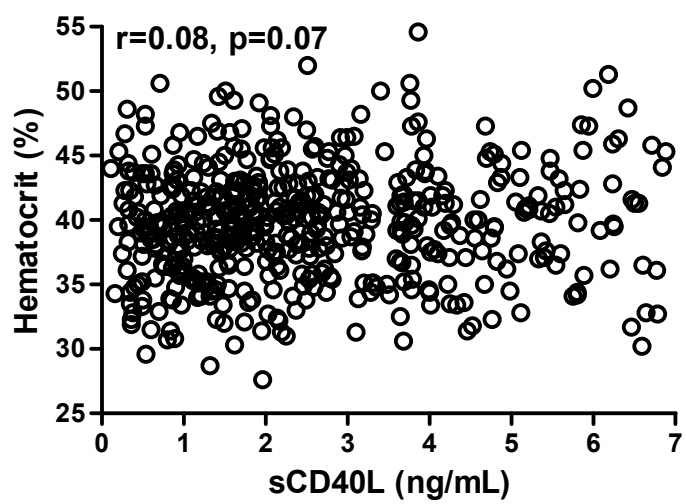

Supplement: S3 Fig — Scatter plot showing sCD40L levels (x-axis) vs. hematocrit (y-axis). Circles represent individual measurements. (PDF) [file pone.0134599.s004.pdf]

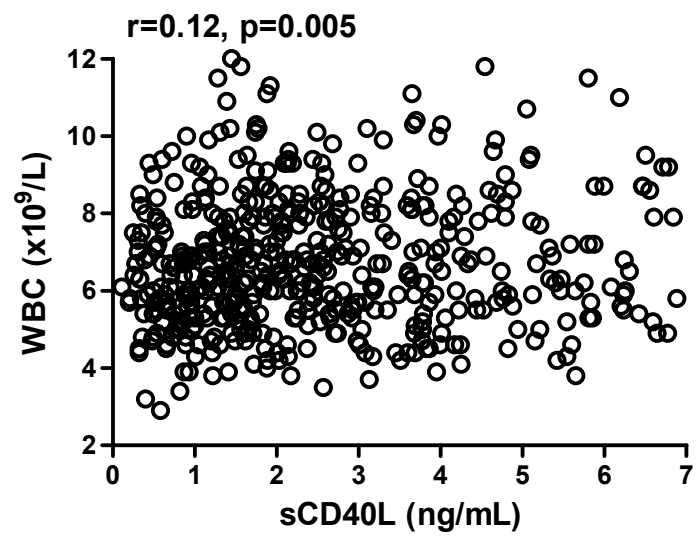

Supplement: S4 Fig — Scatter plot showing sCD40L levels (x-axis) vs. WBC (y-axis). Circles represent individual measurements. (PDF) [file pone.0134599.s005.pdf]

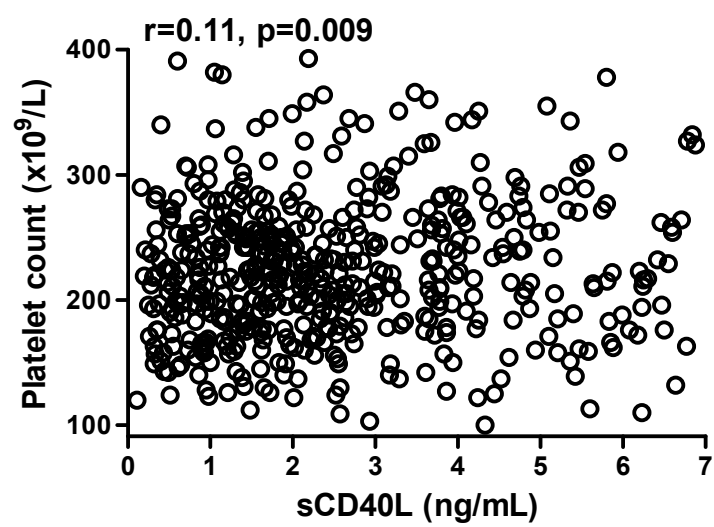

Supplement: S5 Fig — Scatter plot showing sCD40L levels (x-axis) vs. platelet count (y-axis). Circles represent individual measurements. (PDF) [file pone.0134599.s006.pdf]

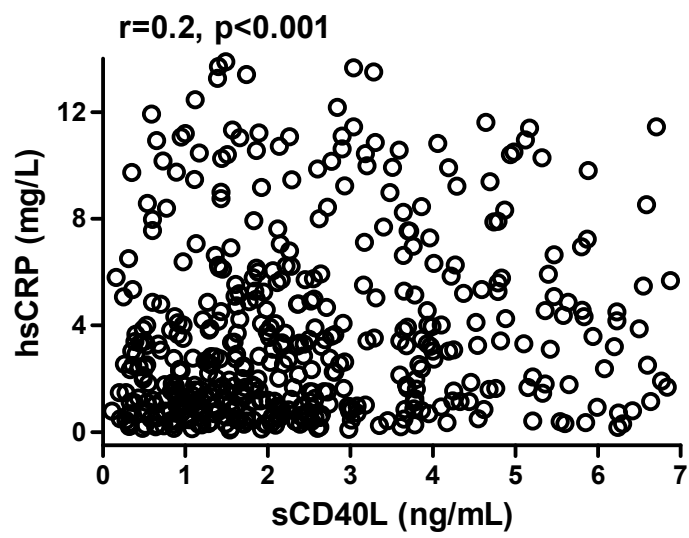

Supplement: S6 Fig — Scatter plot showing sCD40L levels (x-axis) vs. hsCRP (y-axis). Circles represent individual measurements. (PDF) [file pone.0134599.s007.pdf]

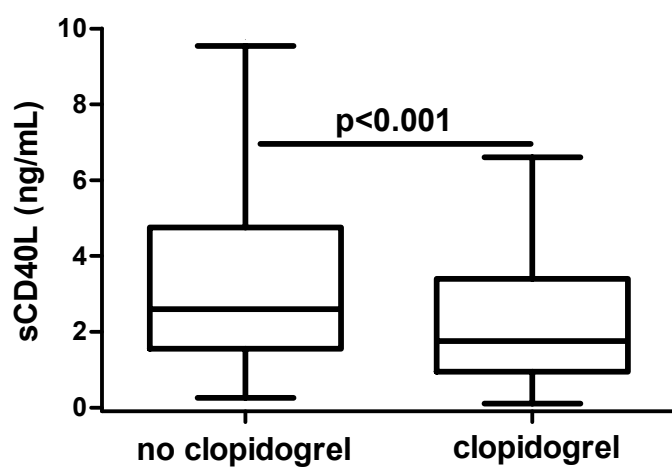

Supplement: S7 Fig — Box plot showing sCD40L levels in patients without and with clopidogrel. The boundaries of the box show the lower and upper quartile of data, the line inside the box represents the median. Whiskers are drawn from the edge of the box to the highest and lowest values that are outside the box but within 1.5 times the box length. (PDF) [file pone.0134599.s008.pdf]

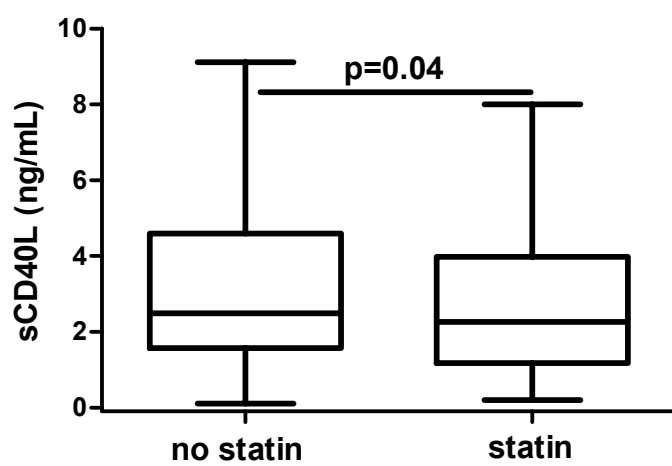

Supplement: S8 Fig — Box plot showing sCD40L levels in patients without and with statins. The boundaries of the box show the lower and upper quartile of data, the line inside the box represents the median. Whiskers are drawn from the edge of the box to the highest and lowest values that are outside the box but within 1.5 times the box length. (PDF) [file pone.0134599.s009.pdf]

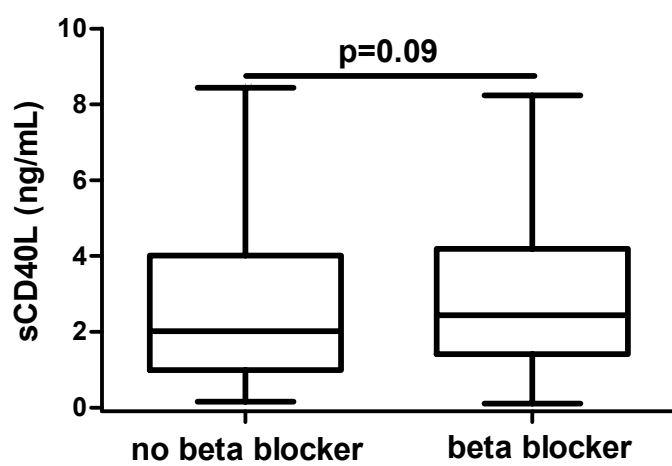

Supplement: S9 Fig — Box plot showing sCD40L levels in patients without and with beta blockers. The boundaries of the box show the lower and upper quartile of data, the line inside the box represents the median. Whiskers are drawn from the edge of the box to the highest and lowest values that are outside the box but within 1.5 times the box length. (PDF) [file pone.0134599.s010.pdf]

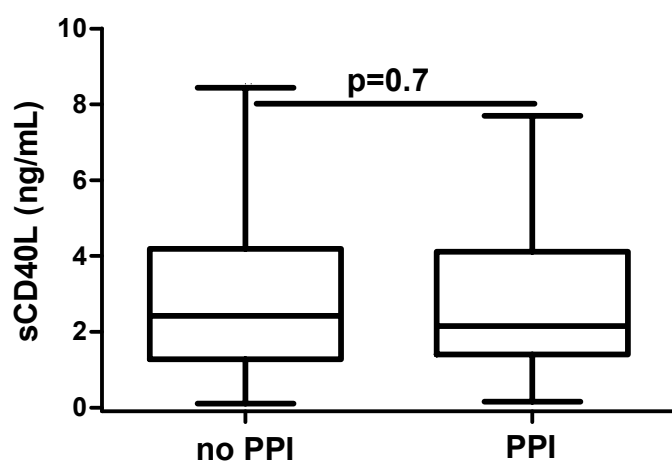

Supplement: S10 Fig — Box plot showing sCD40L levels in patients without and with PPIs. The boundaries of the box show the lower and upper quartile of data, the line inside the box represents the median. Whiskers are drawn from the edge of the box to the highest and lowest values that are outside the box but within 1.5 times the box length. (PDF) [file pone.0134599.s011.pdf]

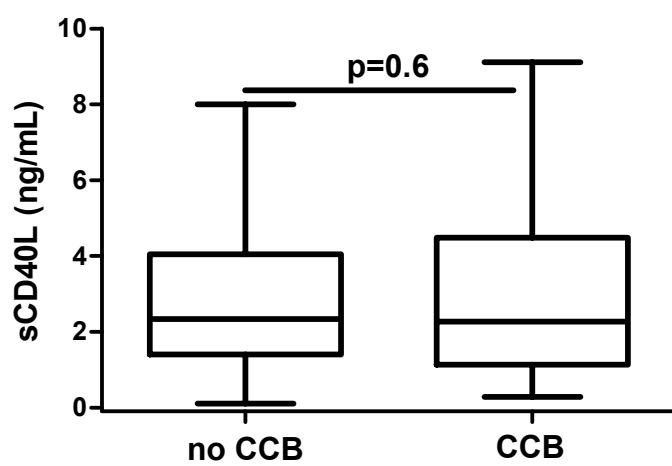

Supplement: S11 Fig — Box plot showing sCD40L levels in patients without and with CCBs. The boundaries of the box show the lower and upper quartile of data, the line inside the box represents the median. Whiskers are drawn from the edge of the box to the highest and lowest values that are outside the box but within 1.5 times the box length. (PDF) [file pone.0134599.s012.pdf]

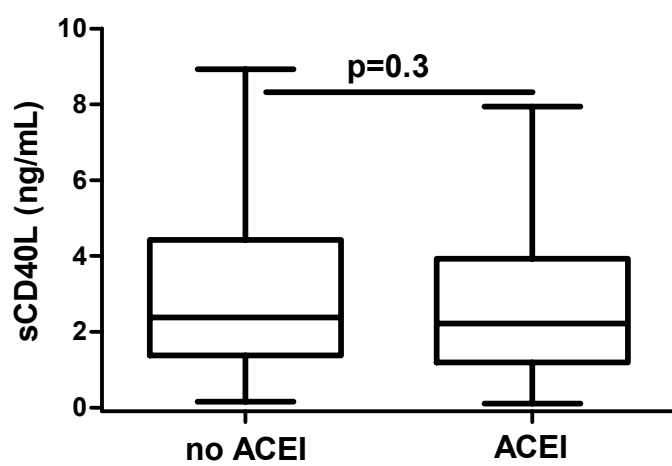

Supplement: S12 Fig — Box plot showing sCD40L levels in patients without and with ACEIs. The boundaries of the box show the lower and upper quartile of data, the line inside the box represents the median. Whiskers are drawn from the edge of the box to the highest and lowest values that are outside the box but within 1.5 times the box length. (PDF) [file pone.0134599.s013.pdf]

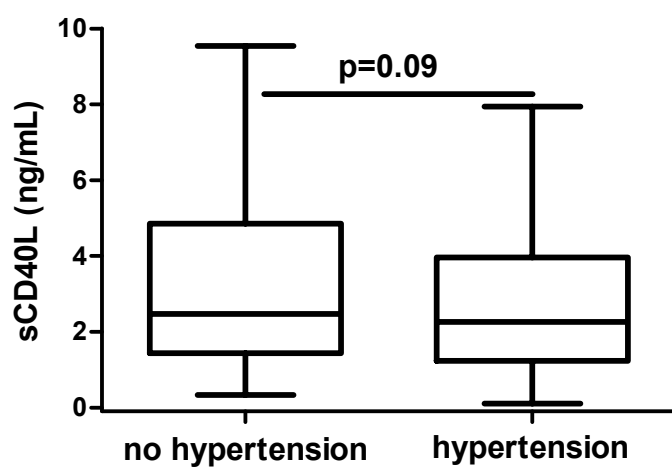

Supplement: S13 Fig — Box plot showing sCD40L levels in patients without and with hypertension. The boundaries of the box show the lower and upper quartile of data, the line inside the box represents the median. Whiskers are drawn from the edge of the box to the highest and lowest values that are outside the box but within 1.5 times the box length. (PDF) [file pone.0134599.s014.pdf]

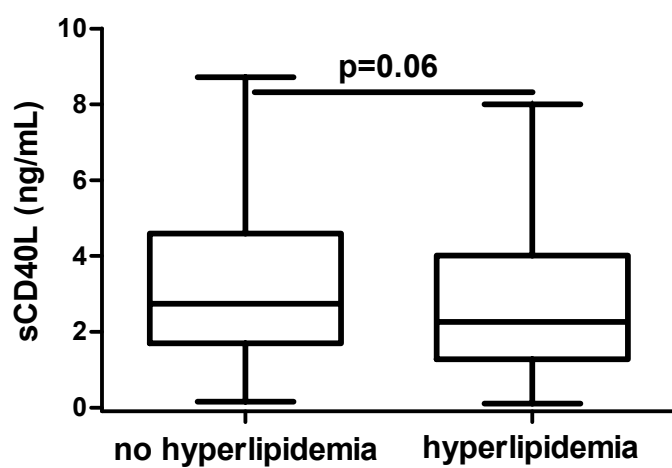

Supplement: S14 Fig — Box plot showing sCD40L levels in patients without and with hyperlipidemia. The boundaries of the box show the lower and upper quartile of data, the line inside the box represents the median. Whiskers are drawn from the edge of the box to the highest and lowest values that are outside the box but within 1.5 times the box length. (PDF) [file pone.0134599.s015.pdf]

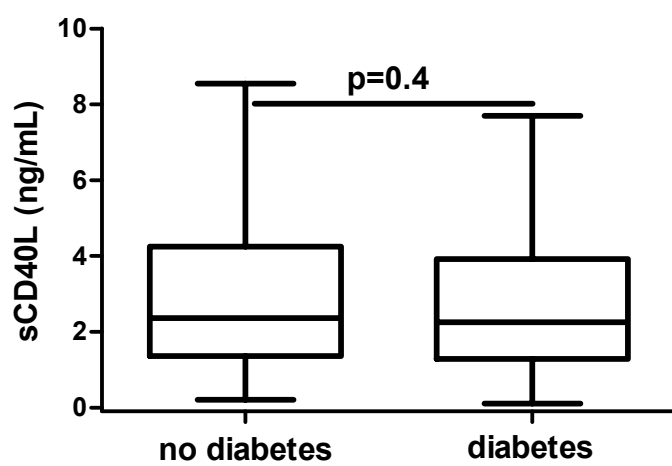

Supplement: S15 Fig — Box plot showing sCD40L levels in patients without and with diabetes. The boundaries of the box show the lower and upper quartile of data, the line inside the box represents the median. Whiskers are drawn from the edge of the box to the highest and lowest values that are outside the box but within 1.5 times the box length. (PDF) [file pone.0134599.s016.pdf]

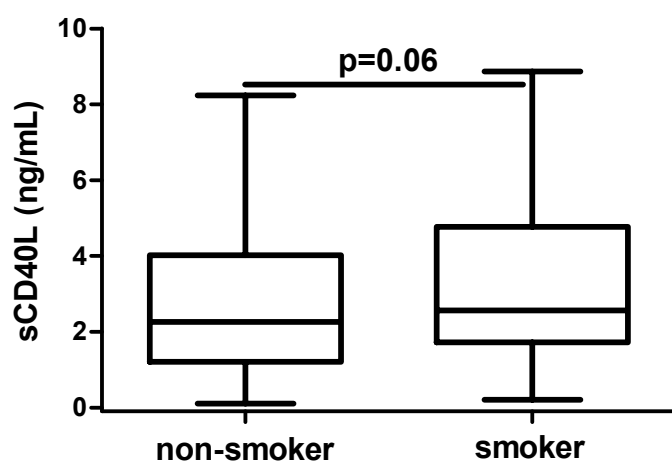

Supplement: S16 Fig — Box plot showing sCD40L levels in non-smokers and smokers. The boundaries of the box show the lower and upper quartile of data, the line inside the box represents the median. Whiskers are drawn from the edge of the box to the highest and lowest values that are outside the box but within 1.5 times the box length. (PDF) [file pone.0134599.s017.pdf]

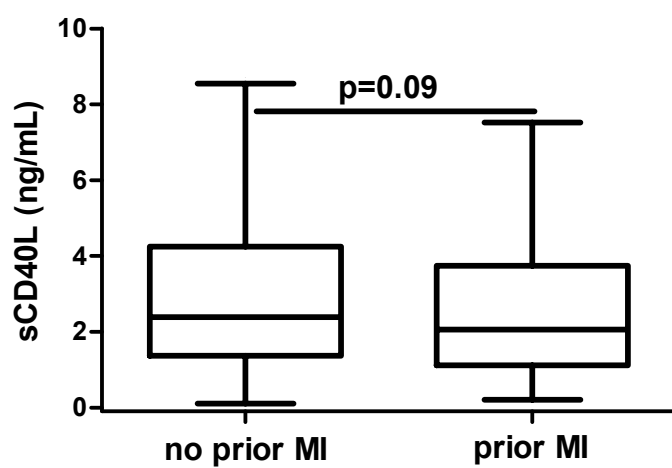

Supplement: S17 Fig — Box plot showing sCD40L levels in patients without and with prior MI. The boundaries of the box show the lower and upper quartile of data, the line inside the box represents the median. Whiskers are drawn from the edge of the box to the highest and lowest values that are outside the box but within 1.5 times the box length. (PDF) [file pone.0134599.s018.pdf]

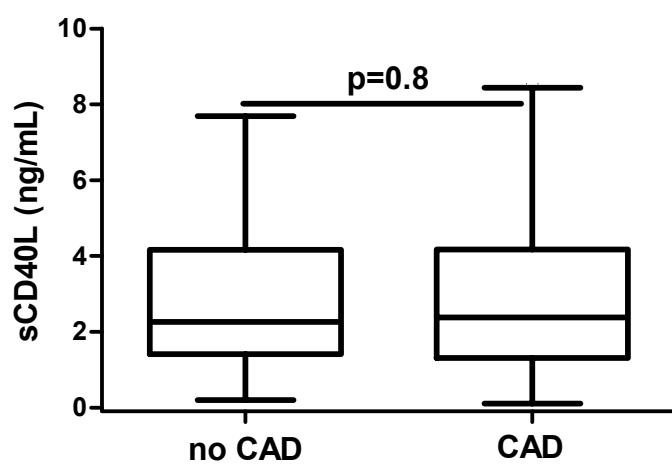

Supplement: S18 Fig — Box plot showing sCD40L levels in patients without and with CAD. The boundaries of the box show the lower and upper quartile of data, the line inside the box represents the median. Whiskers are drawn from the edge of the box to the highest and lowest values that are outside the box but within 1.5 times the box length. (PDF) [file pone.0134599.s019.pdf]

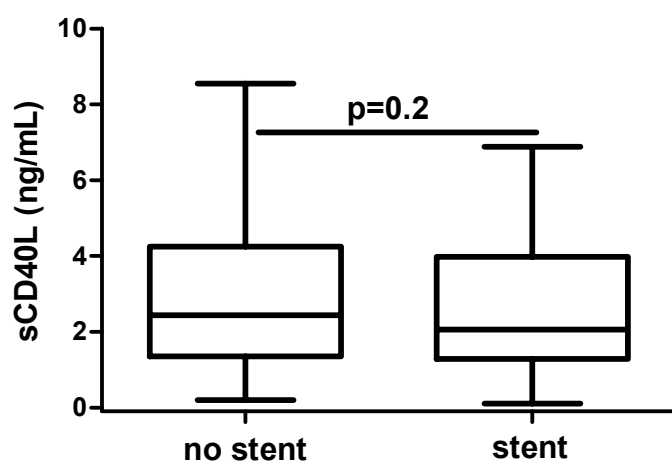

Supplement: S19 Fig — Box plot showing sCD40L levels in patients without and with stent implantation. The boundaries of the box show the lower and upper quartile of data, the line inside the box represents the median. Whiskers are drawn from the edge of the box to the highest and lowest values that are outside the box but within 1.5 times the box length. (PDF) [file pone.0134599.s020.pdf]

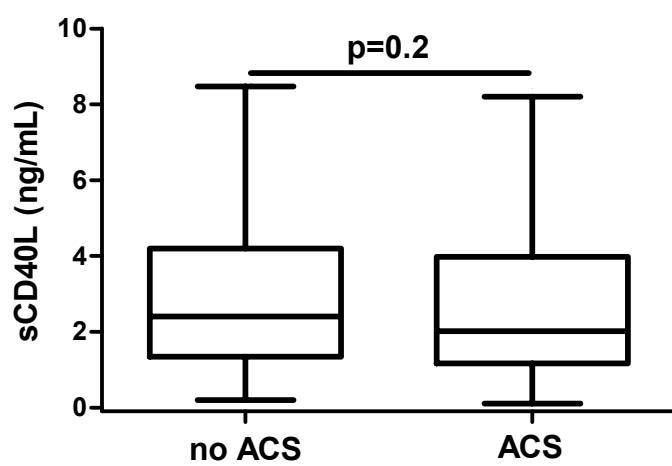

Supplement: S20 Fig — Box plot showing sCD40L levels in patients without and with ACS. The boundaries of the box show the lower and upper quartile of data, the line inside the box represents the median. Whiskers are drawn from the edge of the box to the highest and lowest values that are outside the box but within 1.5 times the box length. (PDF) [file pone.0134599.s021.pdf]

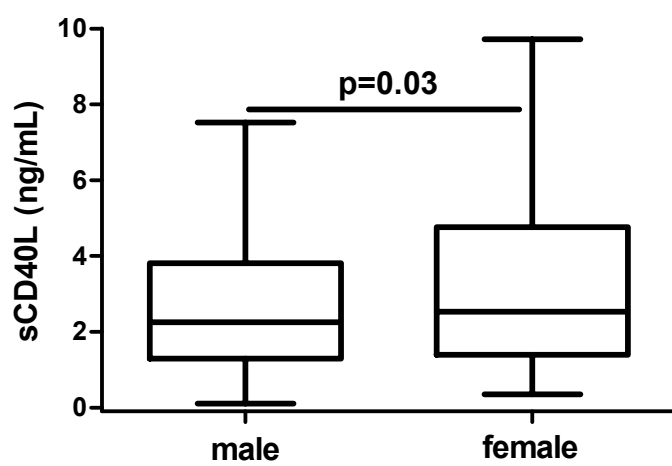

Supplement: S21 Fig — Box plot showing sCD40L levels in male and female patients patients of the study population. The boundaries of the box show the lower and upper quartile of data, the line inside the box represents the median. Whiskers are drawn from the edge of the box to the highest and lowest values that are outside the box but within 1.5 times the box length. (PDF) [file pone.0134599.s022.pdf]

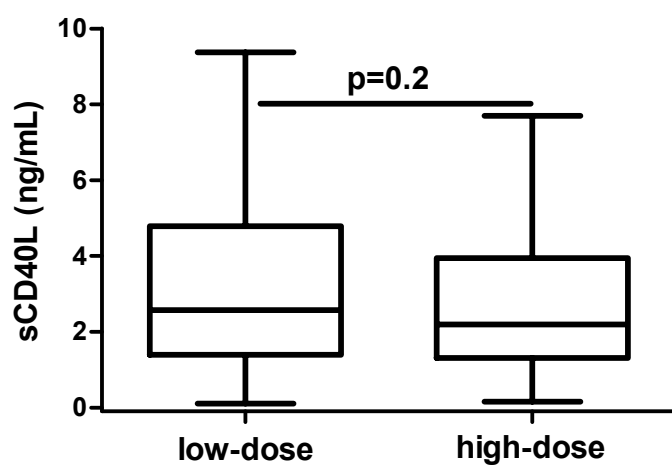

Supplement: S22 Fig — Box plot showing sCD40L levels in patients with low-dose and high-dose aspirin therapy. The boundaries of the box show the lower and upper quartile of data, the line inside the box represents the median. Whiskers are drawn from the edge of the box to the highest and lowest values that are outside the box but within 1.5 times the box length. (PDF) [file pone.0134599.s023.pdf]
